# Editorial: Harnessing natural plant extracts and probiotics to enhance host-gut microbiome interactions

**DOI:** 10.3389/fmicb.2025.1607339

**Published:** 2025-04-28

**Authors:** Kaijun Wang, Chuanshe Zhou, Kang Xu, Jie Yin

**Affiliations:** ^1^College of Veterinary Medicine, Hunan Agricultural University, Changsha, Hunan, China; ^2^Chinese Medicinal Materials Breeding Innovation Center of Yuelushan Laboratory, Changsha, Hunan, China; ^3^Institute of Subtropical Agriculture, Chinese Academy of Sciences, Changsha, China; ^4^Animal Nutritional Genome and Germplasm Innovation Research Center, College of Animal Science and Technology, Hunan Agricultural University, Changsha, Hunan, China

**Keywords:** natural plant extracts, probiotics, gut microbiome, host-microbiome interactions, metabolic health

## Introduction

The intricate relationship between the gut microbiome and host health has emerged as a cornerstone of modern biomedical research. In recent years, natural plant extracts and probiotics have received considerable attention for their potential to modulate gut microbial communities and thereby promote host wellbeing. This Research Topic, “*Harnessing Natural Plant Extracts and Probiotics to Enhance Host-Gut Microbiome Interactions*” brings together 13 cutting-edge studies that collectively advance our understanding of how these interventions can shape host-microbiome dynamics. By exploring mechanisms ranging from immune modulation to metabolic regulation, these contributions underscore the promise of natural compounds in addressing pressing challenges in health and agriculture.

## The gut microbiome as a therapeutic target

The gut microbiome plays a pivotal role in digestion, immune function, and metabolic homeostasis. Dysbiosis—an imbalance in microbial composition—has been linked to diseases such as obesity, inflammatory bowel disease (IBD), and even mental health disorders. Natural plant extracts and probiotics offer a sustainable approach to restoring microbial balance as they often act as prebiotics, antioxidants, or immunomodulators.

In this collection, several studies highlighted the role of plant-derived polysaccharides. For instance, Wen et al. demonstrated that *Atractylodes macrocephala* polysaccharide (AMP) improves growth performance and intestinal health in largemouth bass by enhancing beneficial bacterial taxa such as *Firmicutes* and *Bacteroidota*. Similarly, Zheng Y. et al. showed that grape seed proanthocyanidins (GSP) reduce oxidative stress and promote growth in pigs by increasing the abundance of *Lactobacillus*. These findings align with the growing recognition that dietary polyphenols can act as prebiotics, fostering the growth of health-promoting microbes.

## Immunomodulatory and antioxidant effects

A recurring theme in the contributions is the dual role of natural compounds in reducing inflammation and oxidative stress. For example, Tang et al. revealed that *Rosa roxburghii* polyphenol (RRTP) alleviates acute lung injury (ALI) in mice by enhancing short-chain fatty acid (SCFA) production and increasing *Akkermansia muciniphila*, a bacterium associated with gut barrier integrity. Cheng et al. further emphasized the protective effects of Mulberry leaf polysaccharide (MLP) against cyclophosphamide-induced immunosuppression in chicks, demonstrating improved antioxidant enzyme activity and tight junction protein expression. These results highlight the potential of plant extracts to mitigate oxidative damage and enhance mucosal immunity.

## Metabolic health and disease management

Several studies have explored the impact of natural compounds on metabolic disorders. Freitas et al. investigated the nutraceutical supplement Slim, which reshapes the gut microbiota and reduces *Mucispirillum schaedleri* in obese mice, thereby improving lipid metabolism. Meanwhile, Zheng W. et al. discussed the aryl hydrocarbon receptor (AhR) pathway as a critical mediator of lipid metabolism, linking gut microbial metabolites to host inflammatory responses. These findings underscore the role of the gut microbiome in the metabolic syndrome and suggest that targeted interventions may offer novel therapeutic strategies.

## Probiotics and microbial diversity

Probiotics, live microorganisms with health benefits, are another focus of this collection. For example, Wang et al. demonstrated that supplementation with *Bacillus amyloliquefaciens* alleviates LPS-induced intestinal inflammation in pigs by activating the AhR/STAT3 pathway (Wang et al., 2024). Similarly, studies by Ferrarezi et al. (2024) and Lin et al. (2024) highlighted the potential of probiotics to modulate gut microbial diversity in aquaculture, thereby enhancing disease resistance in fish. These findings emphasize the importance of probiotics in restoring microbial resilience and optimizing host health across species.

## Challenges and future directions

While the studies in this collection provide compelling evidence for the efficacy of natural plant extracts and probiotics, several challenges remain. For instance, the precise mechanisms underlying microbial interactions with host signaling pathways (e.g., AhR, TLR4) require further elucidation. Additionally, standardization of extraction methods and dosage optimization are critical for translating these findings into clinical or agricultural applications. Future research should also explore the long-term effects of these interventions and their scalability across diverse populations.

## Conclusion

This Research Topic consolidates groundbreaking contributions that advance our understanding of how natural plant extracts and probiotics can enhance host-gut microbiome interactions. By addressing inflammation, oxidative stress, and metabolic dysfunction, these studies pave the way for innovative therapies in human and veterinary medicine. As we continue to harness the power of nature, interdisciplinary approaches combining microbiology, immunology, and metabolomics will be key to unlocking the full potential of these interventions.
